# Videoconferencing-delivered psychological intervention for the treatment of COVID-19 related psychological distress in University students: study protocol for a randomised controlled trial in India

**DOI:** 10.1186/s12888-022-04471-4

**Published:** 2022-12-20

**Authors:** Dharani Keyan, Katie Dawson, Suzanna Azevedo, Srishti Yadav, Jasmine Choi-Christou, Deepthi J. Maliakkal, Mohan K. Pillai, Elizabeth Thomas, Tony S George, Richard A. Bryant

**Affiliations:** 1 University of New South Wales, Sydney, Australia; 2 CHRIST (Deemed to be University), Bangalore, India

**Keywords:** COVID-19, Controlled trial, Psychosocial intervention, Psychological distress

## Abstract

**Background:**

The mental health impacts of the COVID-19 pandemic have been profound. This paper outlines the study protocol for a trial that tests the efficacy of a brief group-based psychological intervention (*Coping with COVID;* CWC*)*, relative to Supportive Counselling, to reduce distress associated with COVID-19 in a young adult population in Bangalore, India.

**Methods:**

A single-blind, parallel, randomized controlled trial will be carried out via video conferencing in a small group format. Following informed consent, adults that screen positive for levels of psychological distress (Kessler 10 (K-10 score ≥ 20) and have access to a videoconferencing platform will be randomised to an adapted version of CWC (*n* = 90) or Supportive Counselling (SC) (*n* = 90). The primary outcome will be reduction in psychological distress including anxiety and depression at 2-months post treatment. Secondary outcomes include worry, positive wellbeing, and stress in relation to COVID-19.

**Discussion:**

This treatment trial will assess whether CWC will result in reduced distress relative to Supportive Counselling in a young adult population in Bangalore, India. This study will yield important insights into the role of nonspecific factors versus the intervention’s components in impacting COVID-19 related distress.

**Trial registration:**

This trial was prospectively registered on the Australian New Zealand Clinical Trials Registry (ACTRN12621001064897).

**Ethics and dissemination:**

Ethics approval has been obtained from the participating institution, CHRIST University in Bangalore. Results of the trial will be submitted for publication in peer reviewed journals and findings presented at scientific conferences and to key service providers and policy makers.

**Supplementary Information:**

The online version contains supplementary material available at 10.1186/s12888-022-04471-4.

## Background

A global burden of disease study in 2020 estimated that common mental health problems such as major depression and anxiety increased by at least one quarter during the COVID-19 pandemic [1]. This is not surprising given that the early impositions of social and economic restrictions to curtail the spread of infection around the globe came with vast mental health ramifications. Reports of widespread mental distress including stress, anxiety, depression and hopelessness during the early stages of the pandemic intersected with the uncertainties of such strict containment strategies in addition to the fear of infection itself [2–4]. The mental health impacts have been particularly strongly felt in places with low human mobility and high daily infection rates of COVID-19 [5]. Specifically, low- and middle-income countries (LMICs) posed a particular vulnerability to the long-term mental health sequelae of COVID-19. For example, in India the series of strict lockdowns during the early stages of the pandemic came with the sudden closure of commercial activities and educational institutions across the country. The immediate impact of these restrictions was seen in reduced access to essential services, food insecurities and unstable employment [6, 7]. As the impacts of the pandemic have continued, the mental health costs have persisted in LMICs, including India [8]. To this end, several calls have been made to implement evidence-based interventions in a scalable and readily accessible format, under real-world circumstances, to ward off the potential long-term threats of the pandemic, further widening the gap between availability and demands for quality mental health care [9].

Tertiary students were evidenced to be especially susceptive to the adverse effects of quarantine during the COVID-19 pandemic [10], whereby the prolonged closure of educational institutions took a toll on the wellbeing of young adults [11]. Mental health care that would have been otherwise provided by counsellors based within the universities were likely disrupted as a result of the prolonged pandemic closures. These are pertinent forewarnings given that more than three quarters of the world’s population of young people live in LMICs, where the pandemic continues to have ongoing impacts on economic stress, social isolation and loneliness; all known risk factors for the long-term negative impacts of mental health [2]. Indeed, recent evidence during the COVID-19 pandemic has highlighted the potential oversight of the mental health impacts in young people (aged < 24 years) [9]. Importantly, education settings in low-income settings have a pivotal role in shaping social support networks, protecting vulnerable individual from insecure housing, and in the provision of future opportunities for skilled employment. Naturally, the rise in unintended adverse impacts including depression, anxiety, and worry in young adults [12] is an agenda worth addressing within the broader global mental health strategy.

Scalable solutions to mental health care in response to adversity and crises have been a particular focus in recent years. Problem Management Plus (PM+) is one such intervention developed by the World Health Organization (WHO) [13]. As a low-intensity program, PM + was designed to address common mental health problems arising from adversity in LMICs. The intervention comprises of evidence-based techniques including stress management, problem solving, behavioral activation, and social support enhancement [14]. Developed to be delivered by lay personnel involving those without specialist mental health qualifications, PM + has been shown to reduce mental distress and increase wellbeing in those exposed to adversity and crises across diverse demographic groups [15–19]. Recently, an adapted version of this program was delivered via video teleconferencing, *Coping with COVID* (CWC) was found to reduce worry, anhedonia, and contamination fears arising during the pandemic [20]. In this study, the group-based program addressed the transdiagnostic nature of mental health problems reported during the COVID-19 pandemic and was delivered under circumstances of changing quarantine and lockdowns [20]. To this end, there is scope to test out the efficacy of this adapted version of PM + in LMICs, where the video teleconferencing modality may offer unique benefits for addressing instances of stigma and marginalisation in seeking mental health assistance.

Despite the success of scalable interventions, an outstanding question is clarification of the extent to which treatment gains are attributable to the strategies taught in the programs or to non-specific treatment effects such as counsellor attention or group involvement. All trials of PM + have compared the intervention with usual care, and in doing so have not disentangled these elements. To address this gap, the current study will assess the effectiveness of CWC delivered by lay peer facilitators to target distress resulting from the COVID-19 pandemic in a young adult population and compare this to non-directive group counselling. In using a non-specialist supportive framework involving peers to deliver this evidence-based program, we aim to address potential gaps in access to care, insofar as stigma may act as a barrier in young people utilising evidence-based care. A secondary aim of this study is to assess potential therapeutic impacts of attention and support afforded by peers in driving the effectiveness of CWC. We predict that CWC would lead to greater reductions in common mental health problems including worry, anxiety, depression, and suicidality relative to supportive counselling.

## Methods

### Aims & hypotheses

This study will assess the effectiveness of an adapted version of CWC to specifically target mental distress arising from the COVID pandemic in a cohort of university students in India. The randomized controlled trial (RCT) will compare CWC with Supportive Counselling (SC) in participants reporting moderate to severe psychological distress in a group format. The primary hypothesis is that those receiving group CWC will report lower psychological distress compared to those who received supportive counselling. To this end, we hypothesize that the impact of social support and alliance afforded by the supportive counselling condition will not exceed the therapeutic effects of evidence-based skills covered in CWC. The primary outcome measures are anxiety and depression, as measured by the Hospital Anxiety and Depression Scale (HADS, [21] subscales.

### Design & setting

**Fig. 1:**
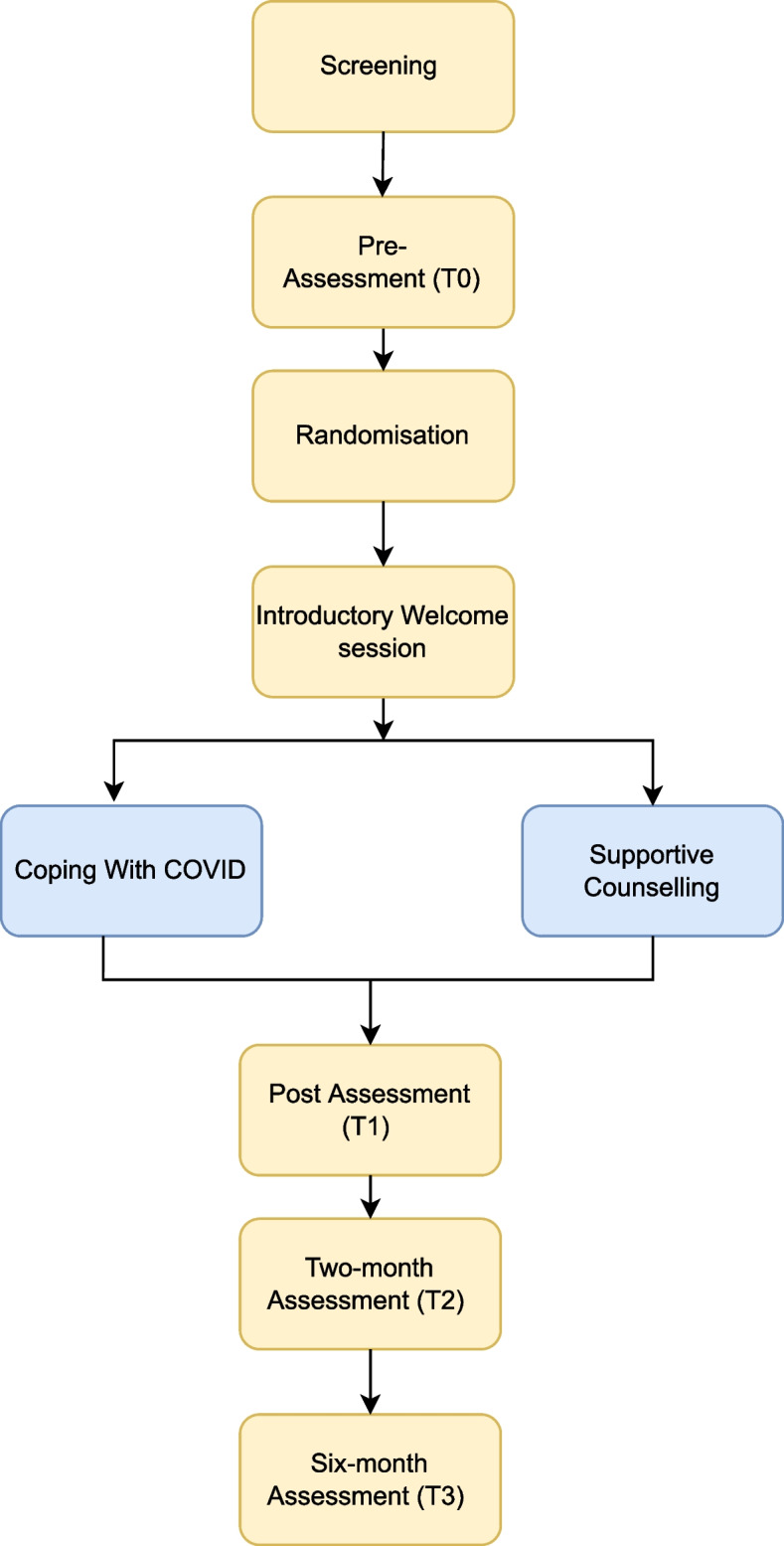
Flow diagram of study

This study is designed as a single-blind, parallel RCT that will be conducted in a university setting in Bangalore, India. The study design will employ a 2 (Treatment Condition) x 4 (Assessment Point) factorial design (see Fig. 1). Participants will be assessed at baseline (T0), post-intervention (T1), and two-( T2) and six-month (T3) follow-ups. The primary outcome time point is set at (T2). This trial was registered on Australian New Zealand Clinical Trials Registry (ACTRN12621001064897) on August 12, 2021 and received ethical approval from CHRIST University in Bangalore, India (ID: CU: RCEC/64/10/21).

Both interventions will be delivered via the Google Meet videoconferencing platform via the CHRIST (Deemed to be University) network that is compliant with the Health Insurance Portability and Accountability Act (HIPPAA). This online platform was chosen as both facilitators and students were familiar with this platform as it was been used for online university classes.

### Participants

Participant inclusion criteria are (a) college-attending young adults (18–25 years); (b) score above 20 on a screening questionnaire for psychological distress (Kessler-10; K-10); and (c) report sufficient English language comprehension. Exclusion criteria are: (a) current psychosis; (b) imminent suicidal risk; (c) current substance dependence (but not abuse); or (d) report of not having access to internet-based teleconferencing facility.

### Recruitment and informed consent

Participants will be identified through university-wide screening via physical advertisements where informed consent will be obtained using a two-step procedure: [1] to conduct screening and [2] to take part in the RCT. Individuals who express interest in taking part in the study will read an explanation of the trial, and then provide informed consent at step one. Those meeting inclusion criteria will proceed to the second step. Those who screen positive will then be sent the T0 assessments.

### Assessment or eligibility and screening

Following receipt of consent, participants will be directed to a webpage that will include the Kessler-10 (K-10); if participants score ≥ 20 on the K-10 they will be directed to complete the T0 assessments. If participants are not selected because they score below the cut-offs for the K-10, they will be provided feedback on their test outcomes and reasons why they are not eligible for the study will be explained to them. If participants meet any of the exclusion criteria, as relevant, participants will be provided with crisis support services, and be provided with external referrals for further management with specialised services including the option to seek support via the internal CHRIST university counselling support service. Pre-assessment (T0) consists of the following instruments to measure primary and secondary outcomes: Hospital Anxiety and Depression Scales (HADS), Generalized Anxiety Disorder Scale (GAD-7), World Health Organization Well-Being Index (WHO-5), Coronavirus Stress Scale (CSS), and Suicidal Ideation Attributes Scale (SIDAS).

### Randomization

The randomization will be conducted by a computerized software on a 1:1 basis and will occur following completion of the T0 assessment. Two project managers who are independent of any other aspects of the treatment study being conducted in Bangalore, India will allocate individuals to CWC or SC groups. Following this time, individuals will be invited for the introductory online session with the group facilitator.

### Introductory online session

After informed consent, and prior to the commencement of groups, participants will be contacted to partake in an initial 15–20-minute introductory online session with a peer facilitator. The aims of this session will be to establish initial rapport between the participant and their facilitator. During this session, participants will also be given basic information relating to how the group sessions will run as well as an explanation of how to use the online teleconferencing platform (Google Meet).

### Interventions

*Coping with COVID* (CWC). The CWC programme is a brief psychological intervention developed to reduce symptoms of stress (e.g., depression, anxiety) for those affected by ongoing adversity. This programme was adapted to include evidence-based strategies that specifically target COVID-19 related distress in a young adult population. This includes education about common reactions to COVID-19, stress management (i.e., slow diaphragmatic breathing), problem solving, worry management, behavioural activation, skills to strengthen access to social support, and relapse prevention. CWC will be delivered over 6 weekly sessions of 60-minute duration. The programme will be delivered in anticipated group size of 4–5 participants via the Google Meet online platform.

*Supportive Counselling (SC).* The SC arm will involve 6 weekly 60-minute video-conferencing sessions that will be led by a trained peer facilitator, who will facilitate group discussions about how students are coping with the pandemic, ventilation of reactions to problems experienced, and discussion of possible solutions. Those randomised to the SC arm will not receive support relating to CWC strategies.

### Screening measure

The Kessler-10 (K-10) is a well validated indicator of psychological distress [22] that will be used to screen individuals eligible to partake in the trial. The K-10 comprises of 10 questions about general wellbeing, experience of emotional states including symptoms of depression and anxiety over the past four weeks. Items are scored on a 5-point scale (range 0 to 50; higher scores indicate higher levels psychological distress), where a cut off of 20 [23] will be used to screen individuals with psychological distress in relation to COVID-19.

The Suicidal Ideation Attributes Scale (SIDAS) will be used to screen the presence of suicidal thoughts and their severity, and is also used as a secondary outcome. This scale consists of 5 items targeting varying attributes of suicidality including frequency, controllability, closeness to attempt, levels of distress associated with suicidal thoughts and consequent impacts on functioning. The SIDAS has demonstrated high internal consistency and good internal validity with GAD-7 and PHQ-9 [24].

### Primary outcome

The primary outcome will be severity of anxiety and depression symptoms as measured using the Hospital Anxiety and Depression Scales (HADS) [25]. The HADS is a 14-item scale consisting of two sub-scales: HADS-A (Anxiety, 7 items, range 0–21) and HADS-D (Depression, 7 items, range 0–21). Higher scores indicate more severe anxiety and/or depression. The minimal clinically important difference (as based on the effect size approach) has been determined at 1.32 for HADS-A and 1.40 for HADS-D and 1.17 for the HADS total score [26].

### Secondary outcomes

The presence of worry symptoms and generalised anxiety will be assessed using the Generalized Anxiety Disorder Scale (GAD-7) [27]. The GAD-7 has demonstrated good reliability, and validity [27] where higher scores indicate more severe symptoms (range 0–21). High levels of sensitivity (89%) and specificity (82%) relative to generalised anxiety disorder has previously been demonstrated using this scale [28]. Further, self-report and interviewer administered versions of this scale have demonstrated good comparability.

Symptoms of stress and anxiety related to COVID-19 pandemic will be assessed using the COVID Stress Scales (CSS) [29]. This scale was developed to index these concerns across 36 items comprising of five domains including 1) COVID danger and contamination fears, [2] COVID fears about economic consequences, [3] COVID xenophobia, [4] COVID compulsive checking and reassurance seeking, and [5] COVID traumatic stress symptoms. This scale had evidenced good reliability and internal consistency [29].

Positive psychological wellbeing will be assessed using the World Health Organization Well-Being Index (WHO-5) as a measure of responsiveness to group CWC or SC. The tool consists of 5 items on 6-point Likert scale, where respondents are asked to rate the extent to which each item related to them over the past 14 days. The WHO-5 has been used to detect aspects of depression in terms of positive mood, interest and energy [30].

All primary and secondary outcome measures have been detailed in Table 1.

**Table 1:** Overview of measures administered at assessment time points

*Concept*	Pre-assessment T0	Post-treatment T1	2-months post-treatment (primary outcome timepoint) T2	6-months post treatment T3
1. Psychological distress	K-10 (**screener**)			
2. Anxiety and depression	HADS (**primary outcome)**	HADS	HADS	HADS
3. Generalised anxiety	GAD-7	GAD-7	GAD-7	GAD-7
4. Wellbeing	WHO-5	WHO-5	WHO-5	WHO-5
5. COVID stress	CSS	CSS	CSS	CSS
6. Suicidal Ideation	SIDAS	SIDAS	SIDAS	SIDAS

### Facilitator selection, training, and supervision

The CWC and SC interventions will be conducted by trained peer facilitators, who will be male and female non-specialist providers recruited from CHRIST (Deemed to be University) campuses across India. They will receive 8 days of training in basic counselling skills, delivery of CWC and SC techniques, group facilitation and self-care. At the end of training, all facilitators will be required to conduct a practise group of both CWC and SC interventions under the close supervision of trained clinical psychologists. In the event that a practice cycle cannot be conducted, a role-play competency assessment will be conducted. To this end, both practice cycles and role-plays will be used to assess facilitator competency prior to implementation of groups for the RCT. Regular weekly supervision will be provided remotely by video teleconferencing with a clinical psychologist (DK, KD, SA, or SY) to ensure treatment adherence and provide ongoing support to facilitators.

CWC and SC groups will be conducted across two cycles from Oct 2021 to Dec 2022 such that training, practice cycles and groups sessions can be run outside of assessment and holiday periods as determined within the CHRIST (Deemed to be university) college semester schedules. Group debrief and feedback sessions will be conducted mid-way and at the end of cycle 1 implementation of sessions such that important learnings can be carried forward to cycle 2 of the RCT.

### Fidelity and blinding

Adherence to the adapted CWC and SC protocol will be ensured by weekly group supervisions including the group facilitators and supervisors. To evaluate treatment fidelity, a sample of 10% of the CWC and SC sessions will be independently rated for adherence. A structured observation form comprising of the presence of the elements pertaining to both CWC and SC programs will be scored by trained members of the research team. Sessions will not be transcribed but raters will make ratings on the basis of audio files that can be accessed by encrypted passwords. To ease the burden of rating of many treatment sessions, different raters will be recruited throughout the course of the trial. Raters will be blind to treatment condition of the sessions they rate, and will be required to nominate what therapy components were present in each session and the quality with which these components were administered.

### Data management and trial monitoring

A trial management committee of the principal investigator, co-investigators, and local research coordinators will monitor the implementation of study procedures on an ongoing basis. Any adverse events that occur during the trial (e.g., increase in distress, suicidal attempts) in a participant that is not related to the CWC intervention, will be recorded by the research team and reported to the study’s trial management committee and Trial Sponsor. These adverse reactions will be referred to appropriate services either within the university counselling service and/or through external psychological support. These services will be nominated by the local clinical psychologist (ET), external to the study, but within the university.

Given the nature of this intervention trial, participants will be required to have their identities known to the implementation staff in order to be contacted for any adverse events and related ongoing care, post and follow-up assessments. All assessment data will be collected from online surveys (administered via the Kobo data collection software) and will be imported into the SPSS statistical analysis database for data management and analysis. Access to assessment data will be restricted to the research assistant team who will be blind to intervention allocation throughout the trial. Further details of data security and storage can be found in ethical protocols, which are available on request. Audio recordings of group sessions will be collected in line with CONSORT requirements for subsequent supervision and assessment of treatment fidelity. The research implementation staff will have access to the data via the UNSW Research Long Term Data Store Interface.

### Sample size

The sample size calculation for this treatment trial was based on a two-group comparison of the primary outcome at the 2-month follow-up timepoint. In order to obtain estimates of parameters, a comparable data set from a recent treatment trial in a high-income setting was used [20]. The power analysis indicated that to achieve a moderate effect size of (0.6) a sample size of 90 participants would be needed per arm to provide power of 0.95 (alpha = 0.05, two-sided); on the expectation that there would be approximately 10% attrition at the 2-month follow-up assessment, it was estimated that a total of 200 participants would need to be enrolled into the study.

### Statistical analysis

Baseline comparability between CWC and SC conditions will be analysed using multiple planned comparisons for continuous measures, and chi-squared tests for categorical variables. Hypothesis testing involving assessment of differential change over time between groups will be assessed using Hierarchical Linear Mixed Modelling (HLM). This HLM statistical method presumes an intent-to-treat framework by allowing the number of observations to vary between participants and thereby effectively handles missing data. For each outcome, linear effects of time of measurement, condition, and condition-by-time interaction will be analysed. Fixed effects parameters for the time of assessment and intervention condition will be tested with the Wald test (t-test, *p* < 0.05, two-sided) and 95% confidence intervals. Cohen’s (d) effect size will be calculated for all analyses. Analyses will focus on the primary outcome (HADS) and secondary outcomes (GAD-7, CSS, SIDAS, & WHO-5) for both CWC and SC, with the main outcome time point being the 2-month follow-up, relative to baseline. To assess for potential bias from attrition, completer analyses will also be conducted using only the data on participants completing the allocated intervention as planned. Two-tailed tests will be reported with a significance level of *p* < 0.05 for all analyses.

## Discussion

This study will evaluate CWC in managing COVID-19 related distress in the sequalae of the COVID-19 pandemic. The trial outlined in this protocol is the first to assess the effectiveness of the CWC program relative to an active control intervention, and in a LMIC setting with young adults in a college-going population. The extent to which the effectiveness of CWC relative to SC is demonstrated, this trial will shed important insights on the active components of this transdiagnostic intervention.

## Supplementary Information

**Additional file 1.**

## Data Availability

Not applicable.

## Abbreviations

CWC: Coping with COVID
CSS: COVID Stress Scales
GAD-7: Generalized Anxiety Disorder Scale
HADS: Hospital Anxiety and Depression Scales
HADS-A: Hospital Anxiety and Depression Scales -Anxiety Subscale
HADS-D: Hospital Anxiety and Depression Scales -Depression Subscale
HIPPAA: Health Insurance Portability and Accountability Act
HLM: Hierarchical linear modeling
K10: Kessler- 10 item version
LMIC: Low- and Middle-Income Countries
PHQ-9: Patient Health Questionnaire – 9 item version
PM+: Problem Management Plus
RCT: Randomized Controlled Trial
SC: Supportive Counselling
SIDAS: Suicidal Ideation Attributes Scale
WHO: World Health Organization
WHO-5: World Health Organization Wellbeing Index
